# Women at high risk of HIV-infection in Kampala, Uganda, and their candidacy for PrEP

**DOI:** 10.1016/j.ssmph.2021.100746

**Published:** 2021-01-30

**Authors:** Rachel Kawuma, Andrew Sentoogo Ssemata, Sarah Bernays, Janet Seeley

**Affiliations:** a MRC/UVRI and LSHTM Uganda Research Unit, Entebbe, Uganda; b School of Public Health, University of Sydney, Sydney, Australia; c Department of Global Health and Development, London School of Hygiene and Tropical Medicine, London, UK

**Keywords:** Sex work, HIV prevention, PrEP, Relationships, Mitigating risk, East Africa

## Abstract

Pre-exposure prophylaxis (PrEP), antiretroviral medication for prevention of HIV-acquisition, is part of biomedical HIV prevention strategies recommended for people at risk of HIV-infection. A decision to take PrEP depends on an assessment of ‘being at risk’ either by an individual, or healthcare provider. In this paper, we draw on the concept of ‘candidacy’ to examine the different ways in which women attending a dedicated clinic in Kampala, Uganda, for women at risk of HIV infection (including sex workers), assessed their suitability for PrEP. We conducted in-depth interviews with 30 HIV negative women up to four different times, to gather information on the motives for taking PrEP, and their life history and daily life. All the women described the relevance of PrEP to mitigate their risk of HIV infection. However, there were challenges to adherence because of alcohol use, irregular working hours and a fear of being seen taking pills that others might assume to be HIV treatment. The ways in which the different women used PrEP and interpreted the place of PrEP in their lives were not solely based on their assessment of protecting themselves during sex work. They also used PrEP to guard against infection from their regular partners, and as a tool to allow them to make more money safely, by having sex without a condom with clients. While eligibility to access PrEP was predicated on the women's being in an ‘at risk’ population group, an incentive to use PrEP was to protect themselves from HIV acquisition from a long-term partner and preserve the ‘trust’ in their intimate relationship by having condom-less sex. Applying a candidacy lens we highlight the complexity in how women experience and present as being ‘at risk’ and query the criteria on which categories of risk and PrEP eligibility are determined.

## Highlights

• Women at risk of HIV infection, assessed their suitability for Pre-exposure prophylaxis (PrEP).
• Challenges to adherence include alcohol use, irregular working hours and a fear of being seen taking pills.
• PrEP was used to guard against infection from their regular partners.
• PrEP was used as a tool to allow them to make more money safely, by having sex without a condom with clients.
• We show the complexity in how women may experience being ‘at risk’ and manage their PrEP use.

## 1 Introduction

Pre-exposure prophylaxis (PrEP), antiretroviral medication for the prevention of HIV acquisition among HIV negative individuals, is part of the comprehensive biomedical HIV prevention strategies recommended by the World Health Organisation (WHO) for people at risk of HIV infection (World Health Organization, 2015, 2016, 2019). In Uganda PrEP was first introduced for key populations - people at high risk of HIV infection - in early 2017 (Djomand et al., 2020; Government of the Republic of Uganda, 2016). Current guidance provides PrEP ‘for HIV negative people at substantial risk of acquiring HIV infection’ (Government of the Republic of Uganda, 2018). There is a note in these guidelines which stresses the suitability for certain groups of people:

‘eligibility [for PrEP] is likely to be more prevalent in populations such as discordant couples, sex workers, fisher folk, long-distance truck drivers, men who have sex with men (MSM), uniformed forces, and adolescents and young women engaged in transactional sex’ (pp. 77–78).

Being recognised as being at risk of HIV infection is an indicator for being eligible for PrEP uptake (Fearon et al., 2019; Hill et al., 2020; Kagaayi et al., 2020; Muhumuza et al., 2021; Nakku-Joloba et al., 2019), a message endorsed by the language of the guidelines. Indeed, HIV prevention cascades often start with the identification of those at risk of becoming infected with HIV (Hargreaves et al., 2016; Schaefer et al., 2019). Yet, as recent research in sub-Saharan Africa shows, the identification of people as being ‘at risk’ as set out in national guidelines, may not be a perception shared by the individuals categorised as belonging to a ‘high risk’ population (Camlin et al., 2020; Hartmann et al., 2018; Warren et al., 2018). Other factors, including a low perceived severity of HIV infection (because of the availability of antiretroviral therapy [ART]), attachment to a sexual partner, current knowledge of partner status and a partner's use of condoms, for example, influenced perceptions of a person's own risk (Hill et al., 2020). The epidemiological classification of being ‘at risk’ is endowed with political power, as it determines the allocation of rationed resources.

A woman's perception of being at risk of HIV infection may be calibrated against other risks posed by engaging in sex work. Djomand et al. (2020) in a review of 35 PEPFAR-supported PrEP programmes, observed that PrEP services were included in national treatment and prevention guidelines in five countries, including Uganda, ‘despite the lack of favourable legal environments to safeguard key populations from violence and discrimination’ (p. 213). It is illegal to sell sex or organise commercial sex in any place (Interactions, 2020) in Uganda in accordance with the Penal Code Act. A number of other laws including the Anti-Pornography Act 2014, NGO Act 2016 and the HIV/AIDS Prevention and Control Act 2014 also have an impact on the rights of sex workers (Human Rights Awareness and Promotion Forum, 2016). Despite these legal hurdles, selling sex is an important means of livelihood in a shadow economy for many women and men in Uganda. The population of commercial sex workers in Kampala was estimated to be between 10,000–15,000 people (Hladik et al., 2017; Schwitters et al., 2015), the variation in number being due to different ways of defining and enumerating ‘sex work’ (Harcourt & Donovan, 2005).

The illegal nature of sex work means working in concealed environments and facing the risk of crime and violence. This contributes to the victimisation, stigmatization and discrimination of persons engaged in selling sex and the ways in which risks to health are managed (Eakle et al., 2019).

When considering whether an individual will take up PrEP, it is not only a question of whether they have access, but also whether they believe they need or want it. In a recent study on the uptake of PrEP in China, MSM were asked the question: ‘Think about your situation, do you believe that you are currently an appropriate candidate for PrEP?’ (Xie et al., 2019). The results suggested that these men assessed their ‘candidacy’ based on the perceived benefits of taking PrEP, the nature of their sexual relationships (for example, not having a long-term partner) and their frequency of HIV testing. While these factors are linked to ‘risk’, Xie and colleagues, the authors of this study, considered that the men's own perception of their PrEP-candidacy was not solely because of ‘risky behaviour’. The authors suggest that it may be important to look beyond risk criteria to support the decision-making of individuals deciding whether or not to take up PrEP. Categorising individuals and groups as ‘most at risk’ may not align with what a person considers to be persuasive reasons for engaging in a service offered to them.

Whether individuals perceive themselves to be candidates for PrEP has been used previously in research looking at the reach of PrEP programmes with populations in the United States and Europe (Dubin et al., 2019; Wilson, Chen, Pomart, & Arayasirikul, 2016), including examining a staging to the uptake of PrEP (a Motivational PrEP cascade) with MSM in the United States (Parsons et al., 2017). The concept of candidacy has been used more broadly in the context of health service utilisation (Adeagbo et al., 2019; Coupland & Maher, 2010; Koehn, 2009; Macdonald et al., 2016; Mackenzie, Gannon, Stanley, Cosgrove, & Feder, 2019; Nkosi et al., 2019). Dixon-Woods et al. (2006) used the concept to investigate the process of accessing health care in the United Kingdom looking not only at the initial decision to access care, but also at how access is maintained by the acceptance into a service and decision-making about subsequent service provision. ‘Candidacy’ used by Dixon and colleagues has been adapted for use in a range of settings to investigate what Mackenzie, Conway, Hastings, Munro, and O’Donnell (2015) describe as ‘the socially constructed process by which individuals identify themselves as candidates for particular conditions and interventions, and subsequently negotiate these candidacies with professionals operating in particular service regimes’ (p. 2). Our use of the concept of candidacy is based on this definition. The concept is also linked to the concept of ‘self-efficacy’ as used by Dodsworth (2012) to describe women for whom ‘sex work is perceived as something about which they have a sense of choice’ (p. 531) and work they were competent to do (Benoit et al., 2018). A woman who chooses to protect herself from HIV infection, who believes in the health-efficacy of the drugs (Sewell et al., 2020) may also reinforce her belief in her self-efficacy and self-esteem through asserting her candidacy for PrEP. We investigate how women in Kampala, engaged in selling sex, identified themselves as a person who would access and use PrEP, as candidates for PrEP.

## 2 Methods

### 2.1 Study setting

We conducted a study using qualitative methods among HIV negative women at high risk of acquiring HIV at the Good Health for Women Project (GHWP) in Kampala, Uganda. The GHWP is a longitudinal cohort in Kampala that was established in 2008 to recruit and follow up women at high risk of HIV and sexually transmitted infections (STIs) for purposes of research and provision of HIV prevention and care services (Vandepitte et al., 2011). Women were identified and recruited from places where sex work took place such as bars and lodges (Mbonye et al., 2013; Vandepitte et al., 2011). Women were eligible for enrolment at the clinic if aged ≥18 years and reported engaging in commercial sex (self-reporting being female sex workers or received money, goods, or other favours in exchange for sex) or working in entertainment facilities and were willing and able to return for follow up visits and study procedures every three months.

In this paper we draw on data from a sub-set of the women enrolled in GHWP who took part in the PRe-Exposure Prophylaxis Priming of Immune Effectors (PREPPIE) study, an observational open label study where women were provided with a monthly supply of Tenofovir Disoproxil Fumarate and Emtricitabine (TDF/FTC, “Truvada®“, Gilead) to be taken daily for HIV pre-exposure prophylaxis (PrEP) for one year. The aim of the PREPPIE study was to evaluate participants’ immune responses to HIV and HIV infection status during the year. The total sample size for PREPPIE was 220 women who were HIV negative at the time of enrolment. In-depth interviews with about ten percent of these women were conducted to gather information on the facilitators and barriers to adherence to PrEP and also covered wider aspects of the lives of the women and how PrEP fitted into that context.

### 2.2 Sampling

Thirty women participating in the PREPPIE study were purposively selected for in-depth interviews. This sampling took place as the overarching study recruitment proceeded, to space the 30 women chosen for this study across the PREPPIE recruitment period. We did not, therefore, wait for all 220 women to be recruited before starting to select women to take part in the in-depth interviews. Care was taken to balance the sample to include women of different ages and those who came from different locations of residence within Kampala to obtain data from women representing the different women who attended the clinic and chose to take part in the PREPPIE study.

### 2.3 Data collection/analysis

We used in-depth interviews to gather information on women's motives for taking PrEP but also on their life history and daily life. Data collection was done over four phases: one, six, 12, and 18 months post enrolment in the PREPPIE trial and first use of PrEP. Thirty women were interviewed during phase one. After analysis of the month one interviews, we purposively followed-up approximately half of the women from this initial phase to participate in subsequent interviews. Women were selected to represent different backgrounds, social contexts and experiences using PrEP as well as continued willingness to share their experiences with the interviewer. The selection was also influenced by availability, because the GHWP population travel frequently for work and other reasons, which results in women missing appointments. Therefore, we interviewed 17 participants at phase two (six months), 11 at phase three (12-months) and nine at phase four (18-months).

Interviews were conducted between August 2017 and October 2019. The repeat interview design was adopted to explore women's experience of PrEP over time, including how they adapted their sexual behaviour as they got used to taking PrEP. An interview guide was developed to facilitate discussion on entry into sex work, knowledge of biomedical HIV prevention methods, motivation to start PrEP, perceptions of HIV risk, experiences using PrEP, changes that came with using PrEP, future aspirations (whether PrEP played a part in these plans). All interviews were conducted in a private space at the clinic by a team of experienced social scientists proficient in Luganda, the local language. The interviews were conducted on days which coincided with the women's designated clinic visits. Interviews lasted 40–60 minutes and were not audio-recorded, an arrangement agreed with GHWP participants to enable the participants to feel more comfortable in sharing their experiences (Rutakumwa et al., 2019). Brief notes were written during the interview and then written up in detail shortly after each interview. Identifying information was not included in the scripts (such as names or precise locations). Pseudonyms are used in this paper.

Members of the social science team reviewed the notes and identified emerging themes from the initial phase of data collection. This information was used to inform subsequent topics for discussion during interviews. Translated interview scripts were double-coded manually using a thematic coding framework, following the general principles of thematic analysis (Braun & Clarke, 2006). Information from each script was charted on to a series of thematic matrices in Excel to provide an overview of the coded data. The five main themes which emerged from analysis are used as subheadings to order our findings. In this paper excerpts from interview scripts are labelled using the pseudonym of the woman and the in-depth interview (IDI) number which corresponds to the phase of interview (for example IDI-2).

### 2.4 Ethical approval

The study received ethical approval from both the Uganda Virus Research Institute Research Ethics Committee (Ref: GC/127/17/03/545) and the Uganda National Council for Science and Technology (Ref: HS1811). All the women provided written informed consent to participate in this qualitative methods study.

## 3 Findings

Out of the 30 women in this study, thirteen were between the ages of 20–39 years. Six participants were above 40 years with the oldest being 52 years old. Twenty-six were single mothers. All the women had had, or still had, regular partners whom they referred to as a boyfriend/husband, these were relationships where there was a lasting bond between the couple. However, few women received support for their children from these partners. In addition to needing to provide for their children, several women had other dependents to look after, including their younger brothers and sisters and their mothers and other older relatives. While 23 of the women had not completed primary school, one woman had studied up to university level but was unable to complete her studies because she did not have money for the fees. The women worked in bars, did hairdressing, sold second-hand clothes or cooked food/vegetables to make money in addition to providing sex for money.

All the women had met the eligibility criteria to join the GHWP, which required they (or a friend who may have introduced them to the clinic) had considered themselves candidates for a clinic providing care and prevention services, as well as conducting research, for women at high-risk of HIV, including sex workers.

Before turning to our data on PrEP uptake and use, we reflect on the ‘push’ (e.g. poverty, neglect, abuse) and ‘pull’ (money, independence) factors which resulted in the women in our study taking up sex work. A decision, which these women often portrayed in the way they recounted their histories, as something about which they had made a choice even when a lack of education and poverty provided few other options.

### 3.1 Taking up sex work

The circumstances under which the women took up earning an income by providing sex for money were very similar. Broken marriages/relationships were common, so too was a childhood clouded by poverty and harsh treatment from relatives (often when their parents had died or had sent them to live with someone else). This meant they missed out on attending school, had a relationship and pregnancy in adolescence and then, when the relationship foundered, needed to make money not only for themselves but also their child. Some women wept as they told the stories of the treatment they had received from husbands and boyfriends. While sisters, brothers, aunts and uncles had often been instrumental in helping them find work, as maids or helping in shops, the money they could make was limited. Nice (aged 21 years) recalled being offered some money by a customer in the bar she was working in to have sex with him, and realising that if she slept with customers she would have ‘a meaningful amount of money’ to send home to her mother, who was caring for her child. Berna (aged 46 years) said she had been excited the first time she received money from a man for having sex, because it was so much more than she was able to make from the kiosk where she was providing access to a telephone (in the days before mobile telephones). Several women mentioned friends or female neighbours who introduced them to selling sex for money. Lucy (27 years old) recalled noticing how friends renting single rooms in the neighbourhood where she stayed in Kampala with an aunt ‘lived a good life’ and had money. When she asked where they worked, one of the women told her and then coached her in selling sex. Lucy said:

“of course I was very nervous but because life was tough and I needed to earn money, I went in and did the work. And besides, my friend was very supportive and was around all the time to ensure that I am safe and no one is taking advantage of me.” (IDI-1)

The example of other women, women like them, was influential in the decision to try to get money from sex. One woman commented that once she started to provide sex for money, she ‘did not look back’. No other livelihood options could provide the same income, even for women who had had secondary school education. Faced with limited options all the women saw themselves as ‘candidates’ for the work of selling sex, they portrayed themselves as having made a choice to take up this work. At the time of this study, 26 of the women were providing sex for money as their main source of livelihood. The four other women used sex when they needed additional income.

The places where the women worked were very fluid as they would change to meet the demands of the client. While most of them were bar-based, once they received clients, the clients would take them to lodges or sometimes to their homes. The women who had permanent rooms in lodges would wait on the streets in the evenings to get clients (Mbonye et al., 2013).

As a result of sustained HIV prevention messaging through media outlets in Uganda as well as health education provided at the GHWP clinic, the women were aware that they were at risk of acquiring HIV. The women commented on the inadequacy of their current prevention methods which exposed them to risks both through paid sex but also within their relationships, therefore PrEP had the potential to offer more effective protection across both domains.

### 3.2 Motivations to take up PrEP: why it might be for me?

Although knowledge of antiretroviral therapy (ART) to treat HIV was common among the women, few knew about the options for using treatment as prevention. Some were aware of Post Exposure Prophylaxis (PEP), but none of them had heard of PrEP before they joined the study. Once they were told about PrEP, it had a multifactorial appeal to the women, not only as an effective form of infection prevention from a trusted source (the GHWP), but also as an aid to higher earnings, as we explain below.

Many of the women had been attending the GHWP clinic for over five years. The care that they received at the clinic was distinctive in its non-judgemental approach, compared to access to care in public clinics, and the women had a high degree of trust in the staff providing care. This influenced their interpretation of PrEP, conferring credibility to it as an option because it was presented to them by trusted healthcare staff.

“I was initially concerned that the drugs may affect and possibly cause me to get cancer but since it was being given to me by health workers, my fears diminished and I felt it may be helpful after all.” (Judith, 39 years old, IDI-1)

The validation of the service by known health workers was, therefore, a critical step in confirming the women's sense of their candidacy for PrEP. The benefits of taking PrEP, as they were presented to the women, emphasised the HIV protection it would confer to them as sex workers. However, to the participants it presented a broader range of benefits, including increasing their earning potential and greater agency to protect themselves from infection within their relationships with their romantic partners.

### 3.3 Trust in the promise of effective prevention in their context of risk

Until they were offered PrEP, the women reported relying, as far as they could, on using condoms as their primary sexual health prevention method. Condoms were described as offering imperfect protection, undermined by both the risk of physical breakage but also the well-documented challenges in negotiating their use. The appeal of PrEP was precisely because it appeared to offset the limitations of being dependent upon condom use.

The power imbalance between clients and the women tended to make negotiating condom use difficult. The women reported that they were unable to negotiate condom use with coercive clients who sometimes would initially agree to use them but tear off the condoms during sex, because they preferred sex without a condom. Imelda (33 years old) describes the precarity of consistent and effective condom use:

“I started working (sex work) on the street when I was 13 years old but if God has protected me this far without being infected with HIV, then God can get angry and I get infected in my old age. If the medicine (PrEP) is there and can protect me from getting infected with HIV I will use it, because condoms break and even men squeeze them and they burst.” (IDI-2)

An additional factor which impeded women from consistently using condoms with clients was that it was very common for them to have consumed alcohol before and while engaging in sex work. They described alcohol as allowing them to work with confidence:

“… whenever I take alcohol, I get sexual arousal, and this makes me enjoy sex with my clients. It also gives me the strength to have sex with several clients in just one night. For example, I can have sex with about three men in just a day when I have taken alcohol otherwise I can't handle having sex with more than one client.” (Pamela - 23 years old, IDI-1)

Although it was used to bolster their ability to engage in the work, it also undermined their capacity to negotiate condom use, as Joy (40 years old) explains:

“Sometimes when we are with these men in the lodges, they refuse to use condoms and you are too drunk to even resist them so if you take this pill then it can protect you against acquiring HIV.” (IDI-1)

As Joy suggests, unlike condoms the use of PrEP was not reliant on others nor was it a prevention method that needed to be employed at the moment of sexual interaction. Importantly though, an additional appeal of PrEP was that it enabled them to protect themselves when having sex with their regular partners (husbands and boyfriends), as well as with their clients. The bond that existed between a woman and her partner commonly precluded her from being able to propose using condoms as a prevention method. However, having unprotected sex with a partner was still considered a relatively high-risk activity as most women presumed that their partner could also expose them to HIV and other sexually transmitted infections. Ciara (30 years old) described the specific risks that existed within her relationship:

“her biggest fear is that she has a boyfriend and they don't use condoms when they have sex yet she has consistently heard that the boyfriend has other sexual partners so she fears that he will infect her with HIV.“(IDI-1)

Even though many women were aware of the risks, the relationship dynamics meant that they were expected to have unprotected sex with their partner, thus the appeal of PrEP did not revolve narrowly and exclusively around their sex work.

There is an additional inherent tension in the protection that PrEP offers. The women considered that PrEP was likely to be more effective than condoms in engaging with their ‘risky’ behaviours. But this belief also demonstrates the emphasis women placed singularly on HIV, as its use on its own would not simultaneously protect them from sexually transmitted infections and unintended pregnancy. Their ‘candidacy’ for PrEP was configured around HIV risk. However, it may also demonstrate the partial protection that condoms had previously afforded in mitigating STI and unintended pregnancy, given that even prior to initiating PrEP, the women explained that condom use had been inconsistent.

### 3.4 Trying it to ‘see’ if PrEP fits for me

Although PrEP was appealing, being in a position to adhere to taking it every day was challenging. Three key factors undermined adherence. The first was the need to keep the pills concealed to avoid their stigmatising association with anti-retroviral therapy, the treatment taken by people living with HIV. The second was the disruptive effects of alcohol on complying with the daily treatment routine. The third, which was often experienced as a temporary impediment, were the side effects from the treatment.

In the context of the women's lives, if someone appears healthy but is taking pills every day, it is a common assumption that they are taking antiretroviral treatment and that they are living with HIV. Although not unusual behaviour, it remains a highly stigmatising association and one that the women in our study were keen to avoid. The fear of being seen taking the drugs, and therefore not being able to take them in the open disrupted their adherence. Although they were taking the treatment to avoid acquiring HIV, they needed to consume their daily pills in secret.

“I hide my PrEP drugs very far where no one can see them because if someone sees them however much you tell them that it protects one from getting infected with HIV, they will not believe you. They will think that you are taking ART.” (Mercy - 24 years old, IDI-1)

“I was somewhere in a leisure place with my friends and the time for taking the pill clocked; I had gone with three pills in a small drug envelope so I asked the waitress to give me water so I bent under the table and took the pill.” (Ciara - 30 years old, IDI-1)

Another barrier to adherence was alcohol. As noted, many women reported relying on alcohol to manage and cope with the varied physical and emotional demands of sex work. Being intoxicated undermined their consistent use of condoms, but it also disrupted their adherence to PrEP as it inhibited their ability to take the pills at a consistent time each day.

“I come back in the morning when I am so drunk and very tired so I sleep it off and wake up at 1pm which means that day passes by without taking.” (Ruth - 43 years old, IDI-1)

Many described having erratic and transient routines because of the nature of the work, which often led them to not returning home and missing daily doses. Mercy (24 years old) when discussing her inability to take the pills daily said:

“The health worker told me to maintain the time which I take these drugs but it so happens that sometimes that time comes when I am not home yet night time is the most convenient for me to take the pills.” (IDI-1)

All the women said they were keen to maintain their adherence to PrEP, but the challenges of their day to day lives presented barriers.

### 3.5 Protective effects of PrEP

When women first began using PrEP they were encouraged by the clinic to use condoms, which they were provided with by the health workers as a parallel prevention strategy. Some of the women explained that they welcomed the option of using condoms because they were not sure PrEP would provide protection, as Zane (27 years old) describes:

“I think it [PrEP] does (protect) but I cannot leave a condom; if it happens and the condom bursts then the drugs can protect me. However, I check every time to make sure that he has properly put on a condom.” (IDI-1)

For those who continued in the study, this however changed over time. As their confidence in the efficacy of PrEP grew so their reported simultaneous use of condoms decreased, with most participants no longer using condoms after twelve months on PrEP.

All but two of the women interviewed at month 12 said that they had stopped using condoms. By being more willing to engage in sex without using a condom, these women reported being able to earn more through each sexual encounter, as such sex (‘live sex’) was more lucrative.

“It is after I started taking PrEP that I started having ‘live sex’. For me if I get a client who gives me a lot of money I go ahead and have ‘live sex.’” (Di - 26 years old, IDI-3)

In this case PrEP can be seen to act in two ways for the woman, while it offers her a sense of protection, she also earns more income by charging a higher levy. Jane (30 years old) explained the amount she could earn if she agreed to condom-less sex was three times what she could earn while using a condom.

“Sometimes I go to the street when I am too broke to even pay for a meal of 5,000/ = [£1]. Imagine if a client comes and offers 70,000/ = [£35] for condomless sex, I wouldn't even think twice to accept because PrEP will protect me from acquiring HIV.” (IDI-3)

Yet, the protective effects of PrEP were limited to preventing HIV. Not using condoms meant that women were exposed to other related risks, including the acquisition of STIs, which three of the women mentioned during the interviews that they were suffering from during the study period. It is important to note that this risk exposure was not characterised as a significant concern to the women in relation to their increased earning power. They emphasised that they had good access to STI treatment through the clinic and combined with their increased earnings, the benefits offset this perceived ‘smaller’ risk.

“Last Friday I came here at the clinic because I had had sex with a man and he had an STI … I didn't immediately notice that I had contracted an STI until after three days and I came here in a lot of pain and was feeling feverish so I came here and saw the clinician and they gave me drugs.” (Zam - 30 years old, IDI-3)

PrEP is considered to provide protection from HIV and it is also economically desirable as it enables women to maximize the value from sex work (sex without a condom). While PrEP use legitimizes low use of condoms, it only offers these women single protection against HIV but not STIs and unwanted pregnancies.

At the end of the study of the nine women interviewed at month 18, six chose to continue using PrEP, because they valued the protection it afforded from, they said, the risk of HIV infection. Two women told us they wanted a break from pills and stopped using PrEP. One woman said she was on treatment for diabetes and felt she was taking too many drugs, so she stopped PrEP.

## 4 Discussion

We set out to investigate the candidacy for women in our study for PrEP. We note above that by joining the GHWP, and thereby asserting their candidacy for the services offered ‘to women at high risk of HIV infection’ (because they fulfilled the clinic eligibility criteria) they were able to access PrEP. Our analysis illuminates the multi-faceted decision-making processes of these women related to PrEP uptake, usage and adherence and how they adjusted in the light of the changing circumstances of their lives and the contexts of HIV risk they navigate. We argue that the classification of being ‘at risk’ is too narrowly determined on a fixed and, commonly, singular criterion which is decontextualized from broader concerns influencing individuals' behaviour. Although participants learnt to deploy a similarly narrow emphasis in portraying their risk status as a means to secure resources, it does not necessarily resonate or adequately explain what influences their decision-making processes in why and how they engage with prevention.

The data we present in this paper show that because the women were familiar with the GHWP, identifying their candidacy, navigating and appearing for the PrEP service, did not pose significant challenges, nor did the adjudication of their eligibility by professionals; all had had their candidacy confirmed by the clinic health workers. However, we suggest that the ways in which the different women used PrEP and interpreted the place of PrEP in their lives, was affected by the different roles they played. Their ‘candidacy’ for PrEP, although underpinned by their recognition of their ‘at risk’ position, and continued engagement with the study, was not solely based on the narrower criterion of being ‘at risk’ i.e. protecting themselves during sex work. The women assessed their candidacy and ‘operating conditions’ for continued PrEP use based on the different roles they performed in their lives: as wives, sex workers and also as research participants.

Most of the women had regular sexual partners; men they called their boyfriend or husband. PrEP allowed them to overcome their concern over acquiring HIV from their regular partners when engaging with sex without a condom; an act which allowed them to portray themselves as a trusting wife or girlfriend. They saw themselves as candidates for PrEP because of their concern over their partner's behaviour, which enabled them to protect themselves while sustaining a valued relationship. The importance of showing trust by having unprotected sex in such relationships has been widely reported (see for example, Okafor, Crutzen, Aduak, Adebajo, & Van den Borne, 2017), including in our own setting (Rutakumwa, Mbonye, Kiwanuka, Bagiire, & Seeley, 2015). The women's use of PrEP was, therefore, not necessarily only because of their engagement in paid sex outside that relationship, but to maximize the beneficial protection it afforded financially and relationally.

However, PrEP use also legitimized a lower use of condoms outside their regular partnership. As we have illustrated, women were able to charge a higher fee for sex without a condom. The additional money they were paid was in recognition of the client's appreciation for what was known as ‘live-sex’, and given the secrecy surrounding PrEP use, few clients, if any, would have known that the woman offering sex was protecting herself from HIV infection. Yet, PrEP only offers these women single protection against HIV but not STIs and unwanted pregnancies. That said, the women were quite ready to disclose their decreased condom use after they had been on PrEP for several months. The women explained that they trusted PrEP and could dispense with condoms. There are additional explanations for the decreased reporting of consistent condom use. Although the sample size at 12 months was small, the pattern emerging from our analysis indicated that using PrEP may legitimise talking about not using condoms consistently. Given that condom-use is commonly over-reported (Liu et al., 2016; Treibich & Lépine, 2019) it may be that rather than their use having changed, they became more confident in revealing lower levels of use. It is also possible that the willingness to acknowledge their lack of condom use, was bound up with their identity as a trusted ‘research participant’ where their reported behaviour was less likely to elicit judgement, even compared to the supportive GHWP staff; the third identity we suggest the women assumed in asserting their candidacy for PrEP.

All the women were established members of the GHWP clinic. All were research participants, familiar with the clinic routine of procedures during their regular visits. The trust which the women expressed about PrEP was, therefore, also likely to be a part of trust in the clinic staff to provide efficacious drugs and to support them when they fell ill with, for example, sexually transmitted infections. In addition, by demonstrating an ability to adhere to PrEP, the women could also show that at the end of the PREPPIE study they were suitable candidates for referral to a PrEP demonstration programme under the Ministry of Health in Uganda, and therefore able to continue receiving PrEP supplies (Nakku-Joloba et al., 2019). Some women opted to stop taking PrEP explaining that they needed a break from taking drugs daily. This suggests that participation in the study was a critical factor in their sustained use of PrEP, rather than being based solely on the women's assessment of their own risk. While this does not suggest that most women believed that their risk of HIV infection was reduced, it may suggest that for some their assessment of risk did not merit sustained use, weighed against concerns of stigma or the burden of fitting pill taking into their schedules. When the need to assert their candidacy for PrEP, based on risk, to stay within the study was withdrawn, some recalibrated their need for PrEP. We argue that understanding their dynamic and active decision-making around whether and when to engage with PrEP, according to their own criteria, further demonstrates their self-efficacy in calculating what was best for them according to their understanding of their personal risk contexts.

### 4.1 Strengths and limitations

The main limitation to this study was that we recruited women already in a setting providing HIV care and prevention services. Therefore, they had substantial knowledge with regards to HIV prevention and were always interacting with the health care workers. This implies that, as much as they had concerns about using PrEP at the start of the study with little knowledge regarding its efficacy, the confidence they had in the health workers encouraged them to use the product. Secondly, the decreasing number of the women participating in the interviews over the four phases reduced the amount of information we had on different women for a longer duration spent taking PrEP and those remaining in this study may reflect a narrower experience, constraining the conceptual generalisability of the findings, although given the range of views obtained, we do not consider that this attrition adversely affected our findings. The benefits the women received as part of participating in this study such as the routine medical care and transport re-imbursement to the clinic every month could have contributed to their motivation to take PrEP. Similarly, face-to-face interviews may have initially prompted social desirability in response, especially related to condom use.

In light of this, the strength of this study was in the methodology used; the use of repeat interviews with the same individual over stipulated time points contributed to the capture of changes in behaviour and perceptions of PrEP, as well as potentially a developing rapport with the researchers which influenced their accounting (Bernays, Paparini, Seeley, & Rhodes, 2017).

## 5 Conclusion

By exploring women's assessment of their candidacy for PrEP, drawing on the work of Mackenzie et al. (2015) and Dixon-Woods et al. (2006), we suggest that, like Xie et al. (2019), we need to look beyond criteria which categorise certain groups as being ‘at risk’ based on their disproportionately high risk of acquiring and transmitting infection including HIV and STIs due to the nature of their work and health seeking behaviours. While access to PrEP was taken up by the thirty women who took part in the study, their reasons for PrEP use did not rest solely on asserting their candidacy based on being a member of a key population, a sex worker. A key incentive to engage in PrEP use was to protect themselves from HIV acquisition, earn additional income by being willing to dispense with a condom and preserve the ‘trust’ in their intimate relationship by having condom-less sex. Applying a candidacy lens, highlights the complexity in how women experience being ‘at risk’ and queries the criteria on which categories of risk and PrEP eligibility are determined. The fact that some women chose to discontinue PrEP at the end of the study may not only be because they wished for a break from pills, but also because they did not consider that the service now offered outside the research context is for ‘people like them’.

## Ethical statement

The study received ethical approvals from both the Uganda Virus Research Institute Research Ethics Committee (Ref: GC/127/17/03/545) and the Uganda National Council for Science and Technology (Ref: HS1811). All the women provided written informed consent to participate in this qualitative methods study.

## Funding statement

This study was funded by the 10.13039/501100000265UK Medical Research Council (MRC) and the 10.13039/501100000278UK Department for International Development (DFID) under the MRC/DFID Concordat agreement and is also part of the EDCTP2 programme supported by the 10.13039/501100000780European Union. JS acknowledges the support of THRiVE-2, a 10.13039/501100000278DELTAS Africa grant # DEL-15-011 from 10.13039/100010269Wellcome Trust grant # 107742/Z/15/Z and the UK government.

## CRediT authorship contribution statement

**Rachel Kawuma:** Writing - original draft, prepared the first draft of the paper. **Andrew Sentoogo Ssemata:** Writing - original draft, prepared the first draft of the paper. **Sarah Bernays:** Writing - original draft, worked on that draft to finalise the paper. **Janet Seeley:** Writing - original draft, worked on that draft to finalise the paper, All authors approved the final version.

## Declaration of competing interest

The authors have no conflicts to declare.
